# In vitro and in silico prediction of antibacterial interaction between essential oils via graph embedding approach

**DOI:** 10.1038/s41598-023-46377-5

**Published:** 2023-11-02

**Authors:** Hiroaki Yabuuchi, Kazuhito Hayashi, Akihiko Shigemoto, Makiko Fujiwara, Yuhei Nomura, Mayumi Nakashima, Takeshi Ogusu, Megumi Mori, Shin-ichi Tokumoto, Kazuyuki Miyai

**Affiliations:** 1 Department of Pharmaceutical Industry, Industrial Technology Center of Wakayama Prefecture, Wakayama, Japan; 2 Department of Digital Manufacturing, Industrial Technology Center of Wakayama Prefecture, Wakayama, Japan; 3 Present Address: Kushimoto Branch, Shingu Health Center of Wakayama Prefecture, Wakayama, Japan; 4 Present Address: Tanabe Health Center of Wakayama Prefecture, Wakayama, Japan

**Keywords:** Cheminformatics, Drug discovery and development, Oils, Natural variation in plants, Secondary metabolism, Antibiotics, Virtual drug screening

## Abstract

Essential oils contain a variety of volatile metabolites, and are expected to be utilized in wide fields such as antimicrobials, insect repellents and herbicides. However, it is difficult to foresee the effect of oil combinations because hundreds of compounds can be involved in synergistic and antagonistic interactions. In this research, it was developed and evaluated a machine learning method to classify types of (synergistic/antagonistic/no) antibacterial interaction between essential oils. Graph embedding was employed to capture structural features of the interaction network from literature data, and was found to improve in silico predicting performances to classify synergistic interactions. Furthermore, in vitro antibacterial assay against a standard strain of *Staphylococcus aureus* revealed that four essential oil pairs (*Origanum compactum*—*Trachyspermum ammi*, *Cymbopogon citratus*—*Thujopsis dolabrata*, *Cinnamomum verum*—*Cymbopogon citratus* and *Trachyspermum ammi*—*Zingiber officinale*) exhibited synergistic interaction as predicted. These results indicate that graph embedding approach can efficiently find synergistic interactions between antibacterial essential oils.

## Introduction

Plants produce and emit diverse volatile organic compounds (VOCs). Humans have found value in the VOCs, and extracted them as essential oils (EOs) by distillation or expression. EOs have been extracted from approximately 3000 plants, and widely used for pharmaceutical, agronomic, food, sanitary, cosmetic and perfume industries^1^. In the last decades, VOCs were elucidated to be involved in protection against pathogens, defense against herbivores, attraction of pollinators and plant–plant signaling^2^. However, it is still uncertain how diverse VOCs cooperatively fulfill their functions under each physiological condition.

Although a large number of EOs and VOCs have been reported to show pharmacological activities^3,4^, development of bioactive products from them is still a challenging task. Many studies have shown that combined EOs exhibit stronger/weaker effects (hereinafter referred to as “EO–EO interaction”) than expected^5,6^. Unfortunately, the causal relationship of the EO–EO interaction is not clear because tens to hundreds of VOCs can be involved in the interaction. Thus, EO products occasionally fail to show the expected activity even though they are generally used in combination.

Advances in machine learning have made significant progress in predicting biologically important pairs such as protein–protein interaction^7^, drug–target interaction^8^ and drug–drug interaction^9^ in the last decades. Traditional approaches represent interaction pair as a numerical vector by operating corresponding (molecular or protein) descriptors, and consider the prediction task as a binary classification problem of the presence/absence of interaction. These classification-based approaches have shown good results for many applications including our previous study on drug–target interaction^10^. However, these approaches are unable to capture complex interactions if the descriptors do not depict characteristics of the interactions. Recently, graph embedding approaches have gained attraction in biomedical fields in order to capture structural features of the interaction network^11^. A systematic comparison on drug–drug interaction showed the graph embedding methods achieved competitive performance without using biological features^12^.

**Figure 1:**
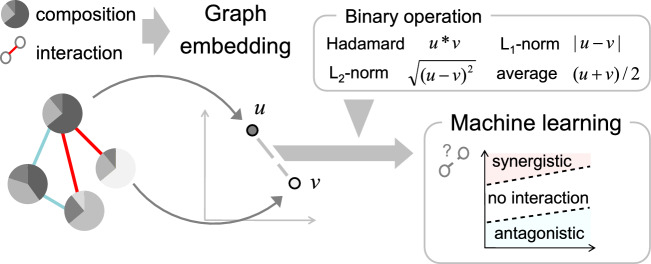
Overview of the graph embedding method to predict interaction between essential oils.

In the present paper, it was developed a machine learning method to predict EO–EO interactions using the graph embedding. The interactions were represented as a network structure with EOs and VOCs as nodes, and their synergistic/antagonistic interactions as edges (Fig. 1). The network structure and oil composition data was integrated to the graph embedding algorithm to encode the nodes as numerical vectors. The edge features were constructed from pairs of the learned node representations with either of binary operators, and were inputted to a machine learning algorithm to classify synergistic/antagonistic/no-interaction pairs. The in silico classification performance was evaluated by cross-validation, a statistical method of evaluating learning algorithms. Furthermore, in vitro antibacterial assay was performed for EO pairs predicted as synergistic by the machine learning model.

## Results

### Graph embedding and machine learning of EO–EO interaction

**Figure 2:**
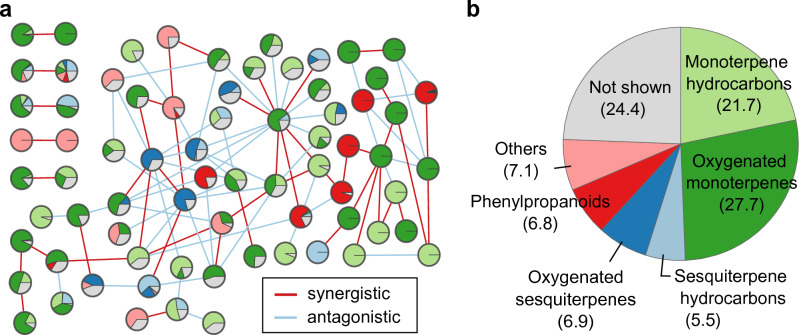
(**a**) Network structure of antibacterial interaction data on *Staphylococcus aureus*. Each edge is colored by synergistic (red) or antagonistic (light blue) interaction. Each node has a pie chart with the chemical composition divided into chemical categories shown in (**b**) for better visualization. (**b**) Mean composition of essential oils in the interaction data. Values in parentheses indicate the mean percentage composition.

The three-class classifier was successfully constructed using graph embedding from antibacterial interaction data composed of 46 synergistic, 53 antagonistic and 172 no interactions between EOs (Supplementary Tables S1 and S2 online). The network structure and chemical composition of EOs were visualized in Fig. 2 for better understanding on the interaction data.

Output probability for synergistic-versus-rest and antagonistic-versus-rest classifications were evaluated by ten-fold cross-validation with receiver operating characteristic (ROC) curve to visualize the relative trade-offs between the true positive rate and false positive rate. Among four (Hadamard, L_1_-norm, L_2_-norm and average) binary operators, average operator showed the best area under the ROC curve (AUC) for both classifications (Supplementary Table S3 online). Furthermore, for the synergistic-versus-rest classification, the operator also showed the best partial AUCs (AUC_0.5_ = 0.211 and AUC_0.2_ = 0.048). Therefore, the average operator was selected for further validation to find unknown synergistic EO–EO interactions. The graph embedding method performed significantly better in AUC (0.615 vs 0.556, *p* = 1.1 × 10^−3^), AUC_0.5_ (0.211 vs 0.164, *p* = 1.7 × 10^−4^) and AUC_0.2_ (0.048 vs 0.033, *p* = 3.8 × 10^−5^) for the synergistic classification than those performed without graph embedding (Table 1). However, no significant differences (*p* > 0.01) were observed for the antagonistic-versus-rest classification.

**Table 1:** AUC and partial AUCs obtained by ten-fold cross-validation.

Classification	Metric	Method	
		Graph embedding	Classification-based
Synergistic-versus-rest	AUC	**0.615 ± 0.020**	0.556 ± 0.040
	AUC_0.5_	**0.211 ± 0.016**	0.164 ± 0.023
	AUC_0.2_	**0.048 ± 0.004**	0.033 ± 0.006
Antagonistic-versus-rest	AUC	0.576 ± 0.014	0.550 ± 0.033
	AUC_0.5_	0.150 ± 0.010	0.159 ± 0.017
	AUC_0.2_	0.020 ± 0.004	0.024 ± 0.003

Values are means ± SD of 10 iterations, and the significantly better results are highlighted in bold (paired* t*-test, *p* < 0.01).

*AUC* areas under the receiver operating characteristic (ROC) curve.

### Prediction of synergistic interaction between available EOs

The probability of synergistic/antagonistic interaction between all possible pairs of the commercially available 84 EOs (Supplementary Table S4 online) were calculated using the classifier constructed above. The classifier predicted 2,088 EO pairs as synergistic when Youden index (= 0.351) was used as the threshold probability. Sixteen EO pairs (Table 2) were randomly selected from them for following gas chromatography/mass spectrometry (GC/MS) analysis and in vitro antibacterial assay.

**Table 2:** Observed antibacterial interaction between essential oil pairs predicted as synergistic.

Essential oil_A_	Essential oil_B_	Probability		MIC_A_	MIC_B_	MIC_mix_	FICI
		Synergistic	Antagonistic	(mg/mL)	(mg/mL)	(mg/mL)	
*C. citratus*	*O. compactum*	0.669	0.075	0.83	0.5	0.5	0.80 (N)
*O. compactum*	*M. balsamum*	0.629	0.161	0.5	> 4	1	1.0–1.1 (N)
*C. citratus*	*M. balsamum*	0.607	0.123	0.83	> 4	2	1.2–1.5 (N)
***O. compactum***	***T. ammi***	**0.585**	**0.190**	**0.5**	**0.5**	**0.25**	**0.50 (S)**
*T. vulgare*	*O. compactum*	0.584	0.182	0.5	0.5	0.5	1.0 (N)
*C. citratus*	*T. ammi*	0.565	0.146	0.83	0.5	0.4	0.64 (N)
*T. vulgare*	*C. citratus*	0.562	0.140	0.5	0.83	0.5	0.80 (N)
*T. ammi*	*L. petersonii*	0.541	0.159	0.5	1	0.5	0.75 (N)
*T. vulgare*	*L. petersonii*	0.538	0.152	0.5	1	1	1.5 (N)
***C. citratus***	***T. dolabrata***	**0.537**	**0.111**	**0.83**	**0.5**	**0.125**	**0.20 (S)**
*C. grevei*	*M. balsamum*	0.535	0.190	1	> 4	> 4	> 2.5 (A/N)
*C. verum*	*O. compactum*	0.504	0.280	0.5	0.5	0.5	1.0 (N)
***C. verum***	***C. citratus***	**0.497**	**0.218**	**0.5**	**0.83**	**0.25**	**0.40 (S)**
*L. petersonii*	*Z. officinale*	0.487	0.132	1	> 4	2	1.0–1.3 (N)
***T. ammi***	***Z. officinale***	**0.415**	**0.285**	**0.5**	** > 4**	**0.25**	**0.25–0.28 (S)**
*T. vulgare*	*Z. officinale*	0.415	0.274	0.5	> 4	0.5	0.50–0.56 (N)

The FICI was interpreted as S: synergistic (FICI ≤ 0.5); N: no interaction (0.5 < FICI < 4); A: antagonistic (FICI ≥ 4). Observed synergistic interactions are highlighted in bold.

MIC_mix_ denotes a summation of two oil concentratations in a mixture. *C. citratus*: *Cymbopogon citratus*, *O. compactum*: *Origanum compactum*, *M. balsamum*: *Myroxylon balsamum var. pereirae*, *T. ammi*: *Trachyspermum ammi*, *T. vulgaris*: *Thymus vulgaris* ct. thymol, *L. petersonii*: *Leptospermum petersonii*, *T. dolabrata*: *Thujopsis dolabrata*, *C. grevei*: *Cedrelopsis grevei*, *C. verum*: *Cinnamomum verum*, *Z. officinale*: *Zingiber officinale.*

### Gas chromatography/mass spectrometry analysis of selected EOs

**Figure 3:**
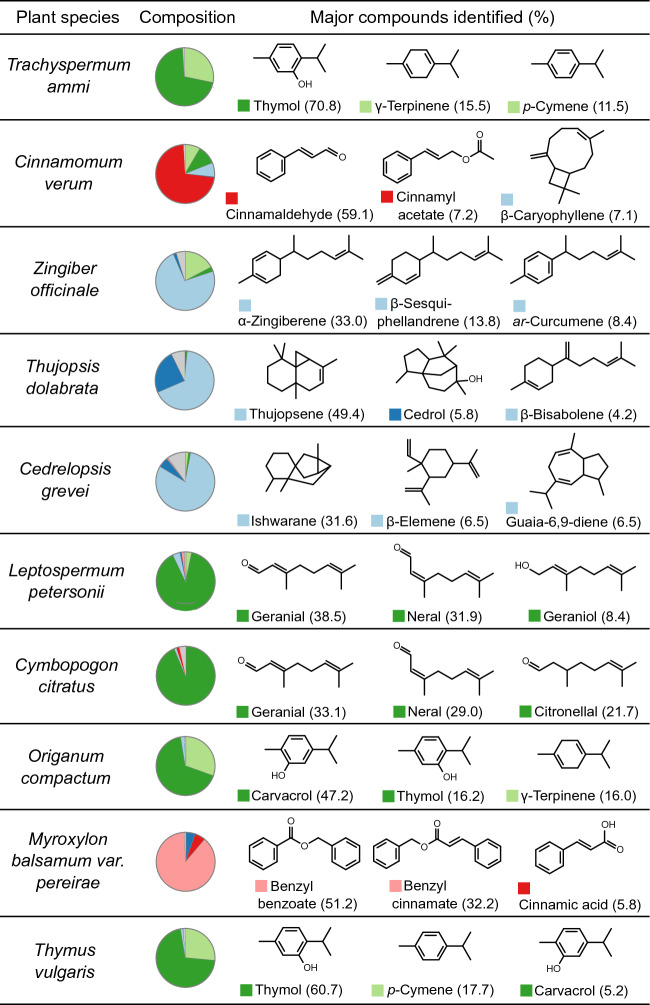
Chemical composition of the selected essential oils. Values in parentheses are the percentage of the total peak area obtained from the total ion current (TIC) chromatogram. Pie charts represent the chemical composition divided into chemical categories shown in Fig. 2b.

In order to obtain more comprehensive composition data for the selected EO pairs, EOs from *Trachyspermum ammi, Cinnamomum verum, Zingiber officinale, Thujopsis dolabrata, Cedrelopsis grevei, Leptospermum petersonii, Cymbopogon citratus, Origanum compactum, Myroxylon balsamum var. pereirae* and *Thymus vulgaris* ct. thymol were analyzed by GC/MS. The most dominant constituents of the EOs were thymol (70.8%), cinnamaldehyde (59.1%), α-zingiberene (33.0%), thujopsene (49.4%), ishwarane (31.6%), geranial (38.5%), geranial (33.1%), carvacrol (47.2%), benzyl benzoate (51.2%) and thymol (60.7%), respectively (Fig. 3). Furthermore, 7, 19, 38, 43, 41, 33, 20, 25, 10 and 21 VOCs from the EOs were respectively characterized by the GC/MS analysis (Supplementary Table S5 online). The classification results of the 16 EO pairs were reproduced by inputting the chemical composition obtained by GC/MS analysis instead of those from EO suppliers.

### In vitro antibacterial assay

Broth microdilution was performed to determine the types of antibacterial interaction between the predicted EO pairs. The EOs and 1:1 mixtures of the EO pairs showed minimum inhibitory concentration (MIC) range of 0.5 to > 4 mg/mL and 0.125 to > 4 mg/mL, respectively (Table 2). MIC for thymol (positive control) was 0.25 mg/mL, which was equivalent to literature data (0.03 v/v %^13^). No inhibition of bacterial growth was observed in the negative control.

Four EO pairs (*O. compactum*—*T. ammi*, *C. citratus*—*T. dolabrata*, *C. verum*—*C. citratus* and *T. ammi*—*Z. officinale*) exhibited fractional inhibitory concentration indices (FICIs) less than or equal to 0.5, namely, synergistic interaction. In particular, the *C. citratus*—*T. dolabrata* combination showed the lowest MIC (0.125 mg/mL) which was lower than that of thymol, and its FICI reached 0.20. Meanwhile, the other 12 pairs showed antagonistic or no interactions.

## Discussion

Artificial intelligence has been applied to classify bioactive EOs using chemical composition data, and has shown good predicting performance^14,15^. However, as far as we know, its application to EO–EO interaction is not yet reported. The difficulty of EO–EO interaction prediction lies in the huge number of chemical constituent pairs to be analyzed compared with the sample number of known EO–EO interactions. In this study, we confronted this problem with graph embedding to compensate the shortage by adding network structure data of the interaction. This strategy worked well for synergistic-versus-rest classification in the cross-validation. The possible reason is that there exists antibacterial contribution of trace constituents absent in the composition data. In fact, several blends of major constituents were known to show much weaker antibacterial activity than original EOs^16^. On the other hand, the graph embedding approach did not show better performance for antagonistic-versus-rest classification in this research. This result suggests that the major components may play key roles in antagonistic actions although the mode of actions is not well known^16^.

The precision obtained by antibacterial assay (4 / 16 = 25%) was apparently low, but the frequency of synergistic interaction should be taken into consideration. It is generally difficult to infer the frequency of EO–EO interactions from the literature data because EO pairs with no interactions tend to be considered as negative results, and to be not reported. An indicative study was performed by Orchard et al*.* testing 247 EO combinations against three reference strains of *Staphylococcus aureus* (ATCC 25923) and methicillin-resistant *Staphylococcus aureus* (ATCC 43300 and ATCC 33592), which resulted in observation of 6, 9 and 14 synergistic interactions, respectively^17^. Assuming that synergism is observed at the same level, our method is expected to detect more synergistic pairs (4 / 16) than random sampling (6 to 14/247).

Predicting interaction against out-of-sample (not learned) EOs is a critical issue because our training data covers just 54 EOs, namely, most of the available EOs lack the interaction data. Furthermore, for each plant species, chemical composition varies under environmental conditions such as temperature, carbon dioxide, lighting and soil fertility^18^. In this study, the graph embedding method successfully detected synergistic interactions for the out-of-sample EOs (*T. ammi*, *T. dolabrata* and *Z. officinale*) and for EOs from different sources (*C. verum*, *O. compactum* and *C. citratus*). This result indicates that the proposed approach is applicable to a wide variety of EOs.

The molecular mechanism of action provides insights to understand the synergistic and antagonistic interactions. Previous studies on EOs pointed out the involvement of hydrophobicity which is responsible for the disruption of bacterial cell membrane^16,19^. For example, carvacrol and *p*-cymene are considered to act synergistically by expanding cell membrane, which results in the destabilization of the membrane^20^. This mechanism may contribute to the synergistic interaction we have found between *O. compactum* (composed of 47.2% carvacrol) and *T. ammi* (composed of 11.5% *p*-cymene). However, the other three interactions (*C. citratus*—*T. dolabrata*, *C. verum*—*C. citratus* and *T. ammi*—*Z. officinale*) are not explained by known interactions between the major constituents. Enrichment of the mechanism information of VOCs will not only provide interpretation of the assay results but also improve the predicting performance of graph embedding approach by incorporating the network structure of VOC–target interactions into the embedding.

Finally, the graph embedding approaches have potential limitations. The first is that the embedding is generally performed in a black-box fashion, which makes difficult to understand which VOCs contribute to the interaction. Feature extraction with wrapper method (*e.g.* recursive feature elimination) may resolve the issue. The second limitation concerns triple or more combination. The method described in this research is based on binary combination for model simplification. Further assay data and statistical theories focused on multiple combination are needed.

Our study suggests that graph embedding approach can efficiently find synergistic interactions between antibacterial EOs. Application of machine learning for other bioactive EO–EO interaction will be evaluated in future research.

## Methods

### Data

Literature search on antibacterial interaction among EOs and VOCs was performed using PubMed^21^ and Google scholar (https://scholar.google.com) in April 2021. The keywords “synergy”, “synergistic”, “antagonistic”, “antimicrobial” and “antibacterial” were used for the search. The tested organisms were restricted to *Staphylococcus aureus*, the most targeted bacteria for exploring antibacterial activity of plant extracts^22^. Cytoscape^23^ (ver. 3.9.1) was used to visualize the EO–EO interaction data.

Chemical composition data of commercially available 84 EOs were retrieved from homepages of product suppliers in Japan. We excluded EOs rich in monoterpene hydrocarbons because their antibacterial effects seemed to be much weaker than other constituents^24^.

### Reagents

Essential oils from *Trachyspermum ammi*, *Cinnamomum verum*, *Cedrelopsis grevei*, *Cymbopogon citratus*, *Origanum compactum*, *Myroxylon balsamum var. pereirae* and *Thymus vulgaris* ct. thymol were purchased from Kenso Igakusha Co., Ltd. Essential oils from *Zingiber officinale*, *Thujopsis dolabrata* and *Leptospermum petersonii* were purchased from TREE OF LIFE Co., Ltd. Acetone for gas chromatography was purchased from KISHIDA CHEMICAL Co., Ltd, Japan. Dimethyl sulfoxide (DMSO) and thymol (special grade, purity 100.0%) were purchased from FUJIFILM Wako Pure Chemical Corporation, Japan. A series of *n*-alkane standards (C_9_ to C_40_) was purchased from GL Sciences Inc., Tokyo, Japan. Mueller–Hinton II broth was purchased from Becton, Dickinson and Company, USA. *Staphylococcus aureus* (NBRC 12,732) for antibacterial activity tests were from the National Institute of Technology and Evaluation, Biological Resource Center (NBRC), Japan.

### Graph embedding

The network structure and oil composition data were inputted to attri2vec^25^, a graph embedding algorithm to encode the nodes as numerical vectors. The number and the size of hidden layer were set to 1 and 16, respectively. Walk length was set to 3, number of walk was set to 3, batch size was set to 32, epochs was set to 50 and learning rate of Adam optimizer was set to 0.01. Binary cross-entropy was chosen as loss function. StellarGraph library (https://github.com/stellargraph/stellargraph) was used for the attri2vec implementation. The edge features were constructed from pairs of the learned node representations with four binary operators (Hadamard, L_1_-norm, L_2_-norm and average)^26^. For comparison with a classification-based method, the oil composition data without graph embedding was used to construct the edge features.

### Machine learning of EO–EO interaction

The edge features constructed above were inputted to multinomial logistic regression with L-BFGS method^27^ to classify the three types (synergistic/antagonistic/no-interaction) of interactions. Output probability for synergistic and antagonistic classes were evaluated by receiver operating characteristic (ROC) curve^28^, respectively. We repeated ten-fold cross-validation 10 times, and used a paired two-tailed *t*-test to determine whether there is any difference in area under the ROC curve (AUC) between the two methods. The partial AUCs were calculated using ‘pROC’ (ver. 1.18.0) R package.

### Prediction of synergistic interaction between available EOs

The probability of synergistic/antagonistic interaction between all possible pairs of the commercially available 84 EOs were calculated using chemical composition data provided by suppliers and the classifier constructed above. Youden index^29^ obtained by the cross-validation was used to set cut-off probability. Sixteen EO pairs were selected for following evaluation. The EOs corresponding to the selected pairs were purchased from the suppliers.

### Gas chromatography/mass spectrometry (GC/MS) analysis

Chemical characterization was performed in the same manner as reported by the authors^30^ using gas chromatograph coupled with mass spectrometer model QP2010 (Shimadzu, Kyoto, Japan). Essential oils were dissolved in acetone (2 μL/mL). This solution (1 μL) was injected in split mode (1:50 ratio) onto a DB-5MS column (30 m × 0.25 mm i.d. × 0.25 μm film thickness, Agilent, USA). The injection temperature was set at 270 °C. The oven temperature was started at 60 °C for 1 min after injection and then increased at 10 °C/min to 180 °C for 1 min, increased at 20 °C/min to 280 °C for 3 min followed by an increase at 20 °C/min to 325 °C, where the column was held for 20 min. Mass spectra were obtained in the range of 20 to 550 m/z. Essential oil components were identified based on a search (National Institute of Standards and Technology, NIST 14), the calculation of retention indices relative to homologous series of *n*-alkane, and a comparison of their mass spectra libraries with data from the mass spectra in the literature^31,32^.

### In vitro antibacterial assay

The essential oil alone and the 1:1 combinations were tested using the broth microdilution assay in the same manner reported by the authors^30^. A stock solution of each essential oil (dissolved to a concentration of 40 mg/mL in DMSO) was diluted to 4 mg/mL by Mueller–Hinton II broth medium, followed by serial dilution by the medium to lower concentrations (2, 1, 0.5, 0.25, 0.125, 0.0625, 0.0313, 0.0156 and 0.0078 mg/mL). Thymol, a known antibacterial agent, was dissolved and diluted in the same way to ensure microbial susceptibility (positive control). The oils were all tested in triplicate. *Staphylococcus aureus* NBRC 12,732 was inoculated onto normal agar plates, and cultured for 24 h at 35 ± 1 °C. The bacterial suspensions were diluted by saline to obtain 0.5 McFarland turbidity equivalent (*ca*. 10^8^ colony forming units per mL (CFU/mL)), and were further diluted 10 times (*ca.* 10^7^ CFU/mL). 0.1 mL of essential oil-containing medium and 5 μL inoculum were added to sterile micro-titre plates. 10% (v/v) DMSO in the medium was used to determine if the solvent exhibited any antibacterial effect (negative control). The micro-titre plates were incubated for 18 to 24 h at 35 ± 1 °C. Based on the opacity and color change in each well, the lowest concentration capable of inhibiting the growth was determined as minimum inhibitory concentration (MIC).

The type of interaction was determined using fractional inhibitory concentration (FIC), a widely accepted means of measuring the interactions^33^, followed by calculating FIC index (FICI) through the equations below:

$${\text{FICI }} = {\text{ FIC}}_{{\text{A}}} + {\text{ FIC}}_{{\text{B}}} ,$$

where

$${\text{FIC}}_{{\text{A}}} = {\text{ MIC}}_{{\text{A}}} \left( {{\text{combination}}} \right)/{\text{MIC}}_{{\text{A}}} \left( {{\text{alone}}} \right),$$

and

$${\text{FIC}}_{{\text{B}}} = {\text{ MIC}}_{{\text{B}}} \left( {{\text{combination}}} \right)/{\text{MIC}}_{{\text{B}}} \left( {{\text{alone}}} \right).$$

The FICI values were interpreted as follows:

$$\le \,0.{5}\, = \,{\text{synergistic}};\,0.{5}{-}{4}.0\, = \,{\text{no}}\,{\text{interaction}};\, \ge \,{4}.0\, = \,{\text{antagonistic}}.$$

### Supplementary Information

Supplementary Table S1.

Supplementary Table S2.

Supplementary Table S3.

Supplementary Table S4.

Supplementary Table S5.

Supplementary Table S6.

## Data Availability

Python scripts are available at https://github.com/yabuuchi-hiroaki/graph-embedding-eo-eo-interaction. All other relevant data are within the paper and its Supplementary Information files.
